# Evaluating the Impact of Digital Health Literacy on the Adoption of Preventive Health Measures in Socioeconomically Vulnerable Communities: A Narrative Review

**DOI:** 10.7759/cureus.94276

**Published:** 2025-10-10

**Authors:** Piyush Kumar Gupta, Keerti Jogdand, Pravin Yerpude, Mohammed Hameeduddin Haqqani, Muhammad Abdulrahman Suheb, Naresh Sen

**Affiliations:** 1 Community Medicine, Geetanjali Institute of Medical Sciences, Jaipur, IND; 2 Community Medicine, Chhindwara Institute of Medical Sciences, Chhindwara, IND; 3 Engineering, Career Point University, Kota, IND; 4 Health, University of New Brunswick, Saint John, CAN; 5 Cardiology, Rama Medical College and Hospital, Kanpur, IND

**Keywords:** digital health literacy, health equity, preventive health behavior, socioeconomic disparities, vulnerable populations

## Abstract

As digital platforms become central to healthcare delivery, digital health literacy (DHL), the ability to seek, understand, evaluate, and apply health information using digital technologies, has emerged as a vital determinant of preventive health behavior. This review addresses how limited DHL hinders the adoption of preventive measures such as vaccination, screening, hygiene, and lifestyle modification in socioeconomically vulnerable populations. The objective is to synthesize quantitative evidence linking DHL to preventive behavior while analyzing the influence of socioeconomic status, digital access, trust in technology, and health self-efficacy. Through a structured review of quantitative studies published between 2015 and 2025, the analysis focuses on validated DHL instruments, including the eHealth Literacy Scale and the Digital Health Literacy Instrument, as well as diverse intervention formats and population-specific findings. Results indicate that improved DHL correlates with up to a 25% increase in preventive health uptake. However, disparities in digital access, foundational literacy, and cultural alignment limit these benefits in underserved settings. Methodological inconsistencies and gaps in region-specific data also constrain generalizability. The findings underscore the need to embed DHL within national health literacy strategies, public health infrastructure, and community-led programs. The key takeaway is that without DHL, digital innovation may widen rather than reduce existing health inequities.

## Introduction and background

Digital health literacy (DHL) has now emerged as the key to public health in the digital age, which involves the capacity to find, interpret, assess, and utilize health information retrieved via digital networks to make decisions [1]. It is a combination of cognitive and social skills that enable people to evaluate the credibility of online health resources and take proper actions [2]. DHL has been identified by WHO as a key to universal health coverage, especially as health systems gradually embrace mobile health (mHealth) technologies, telemedicine, and the use of AI to prevent and treat conditions and illnesses [3]. With increased implementation of these digital tools to deliver healthcare, DHL is becoming a critical social determinant of health, particularly among groups that experience various types of marginalization.

Immunization, screenings, hygiene, and nutrition counseling are often considered effective measures to prevent diseases and, therefore, reduce the burden of diseases [4]. Their adoption does not, however, depend only on the availability of services but also on individuals in terms of awareness, motivation, and ability to make decisions. Digital platforms provide a scalable means of delivering health content in communities with little to no formal health education and inconsistent outreach [5]. Nevertheless, the usefulness of such platforms depends on whether the users possess the DHL that is necessary to interpret, assess, and transfer the information into their context [6].

The concept of the digital health divide brings to light the multiplicative effect of being socioeconomically vulnerable and unable to access healthcare, as well as the digital skills required to take advantage of online health innovations [7]. Individuals in low-income households, with low education levels, living in rural areas, or in social isolation are often left out of the same digital solutions that are supposed to increase equity [8]. These are structural inequalities, meaning that they are part of larger systems that prevent health empowerment and contribute to inequality. Research in high-income countries (HICs) and low- and middle-income countries (LMICs) has repeatedly demonstrated that digital access and DHL are socioeconomically stratified, directly affecting the ability to use preventive health behaviors [9].

The COVID-19 pandemic spurred the world to move to digital public health approaches and highlighted the impact of inequitable DHL. Digital platforms played an essential role in the fight against misinformation, guidelines, and vaccination promotion during the crisis [10]. In digitally literate populations, these interventions were largely effective. Nevertheless, in communities where digital access was low and DHL was low, the outcomes tended to be suboptimal. These populations were more susceptible to misinformation, misinterpretation, and discrepant compliance with health advice [4]. The pandemic thus revealed not only a public health crisis but also severe weaknesses in the digital health system in the absence of explicit attention to equity [11].

**Figure 1:**
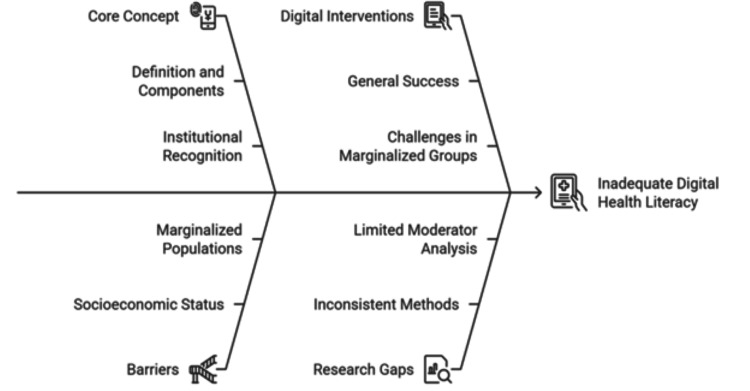
Factors contributing to inadequate DHL among socioeconomically vulnerable populations DHL, digital health literacy Figure created by the authors in collaboration with Napkin AI

Short message service (SMS) reminders, risk calculators, wearable devices, and mobile applications are digital interventions that are effective in influencing preventive health behavior among the general population [12]. Yet, among marginalized communities, their success has been inconsistent. Such discrepancy has frequently been attributed to a poor fit between the intervention design and the linguistic, cognitive, or cultural actuality of the users, and insufficient DHL [13]. Although general health literacy studies are growing, more dedicated studies of the specific effects of DHL on quantifiable preventive health outcomes in disadvantaged groups are rare [14]. The multidimensional factors that contribute to poor DHL are depicted in Figure 1, highlighting key concepts, digital interventions, socioeconomic barriers, and inconsistencies in research done on marginalized populations.

The existing literature on DHL and preventive behavior is quite heterogeneous in terms of methodological orientation and topic. Quantitative research uses diverse measurement instruments, including the eHealth Literacy Scale (eHEALS) and Digital Health Literacy Instrument (DHLI), to measure relationships between DHL and health behaviors, including vaccination, cancer screening, sanitation, and dietary compliance [15]. Although this is broad, the absence of standardized definitions, variability in operationalization of DHL, and the differences in demographic focus hinder cross-study comparability. In addition, the results are also limited in their applicability due to the absence of attention to moderating variables such as gender, accessibility of devices, and the level of digital trust [16]. Such methodological gaps undermine the creation of scalable and inclusive public health strategies sensitive to the barriers that are unique to socioeconomically vulnerable communities.

It is therefore necessary to conduct a focused review of quantitative studies to provide a systematic way of explaining how DHL is related to preventive health behavior. This kind of evidence synthesis has great academic importance in the enhancement of theoretical models that provide a connection between health literacy and behavioral outcomes and the provision of practical knowledge to design culturally sensitive and digitally inclusive interventions. Policy-wise, this research question contributes to the global equity in health through the understanding of how digital innovation can be applied to serve, not to bypass, underserved populations. The fact that the analysis is framed in the context of prevention, as opposed to treatment, also fits the priorities of public health, which recommends upstream disease reduction and health promotion. There is growing evidence that preventive measures informed by well-timed, accurate, and contextually relevant digital content can greatly decrease morbidity, mortality, and long-term health expenditures. But the achievement of these gains is dependent on the population's capacity to interact meaningfully with digital content. This review critically analyzes that relationship, which will inform future studies, help to guide policy formulation, and build the bases of equitable digital health systems.

Objectives of the review

This review aims to evaluate and synthesize quantitative studies examining the relationship between DHL and the adoption of preventive health measures among socioeconomically vulnerable populations. Specifically, it seeks to identify the tools and methodologies used to measure DHL, analyze statistically significant links between DHL and preventive behaviors, examine contextual moderators such as income, education, and access, and highlight gaps that inform future digital public health interventions.

Methodological considerations

This review adopted a systematic methodology to identify and synthesize quantitative studies examining the relationship between DHL and the adoption of preventive health measures among socioeconomically vulnerable populations. A systematic literature search was conducted across PubMed, Scopus, Web of Science, and Google Scholar for peer-reviewed articles published between 2015 and 2025. The search strategy combined relevant keywords and indexing terms, including “digital health literacy,” “eHealth literacy,” “preventive health behavior,” and “vulnerable populations.” Eligible studies used a quantitative design, applied validated DHL instruments (e.g., eHEALS and DHLI), focused on disadvantaged groups (e.g., low income, limited education, and rural settings), and assessed at least one preventive health behavior. Qualitative studies, those lacking DHL measurement or unrelated to prevention, were excluded. Screening and data extraction were conducted independently by two reviewers. Limitations included potential language bias due to English-only inclusion, publication bias, and methodological heterogeneity. Despite these, the review methodology enables a rigorous and replicable synthesis aligned with high-impact standards.

## Review

Conceptualizing DHL

DHL has been developed as a seminal concept in the digitalization of the public health sphere, which reflects the capacity to find, comprehend, assess, and utilize health information delivered by digital resources to form rational health choices [16]. Based on the intersection of traditional health literacy and digital skills, DHL is the response to the information ecosystem that the internet, mobile applications, telemedicine, and AI-based health tools have created [17]. It is multidimensional as opposed to general health literacy, which involves mostly printed or face-to-face communication of information.

The theoretical background of DHL is based on the eHealth literacy model by Norman and Skinner, who combined six literacies, such as traditional, information, media, health, scientific, and computer literacy, to define the ability to communicate with electronic health information [18]. Based on the classification of health literacy by Nutbeam, DHL has been further divided into three gradual levels: functional, communicative, and critical DHL [19]. Basic reading and digital navigation skills constitute functional DHL; the capacity to interpret and use digital health content in different formats is communicative DHL; and critical DHL is the ability to evaluate the reliability, bias, and relevance of information to individual health requirements [20]. Compared to general health literacy, DHL necessitates users to deal with elaborate interfaces, shifting content, and digital risk spaces that are influenced by algorithms and misinformation [21]. As an illustration, using a vaccination portal or analyzing contradictory information about diets on social media requires not only technical skills but also critical thinking. Therefore, DHL entails abilities that are also becoming essential in preventative health behaviors, especially as healthcare delivery and communication become digital-first [22].

Further, DHL is not a fixed skill but a skill that is influenced by the environment of users, such as infrastructure, language context, and cultural trust toward digital sources [10]. An individual can be a competent general literate but weak at DHL because of either having little access to technology or having low digital confidence, an issue that is especially prevalent among older adults, people in rural areas, and those of lower socioeconomic status [23]. The understanding of DHL as an individual and structurally mediated capability is vital in the realization that it helps or hinders access to preventive services. DHL does not just affect information acquisition but has a direct effect on preventive health decision-making, including vaccine acceptance and screening participation [24]. In order to effectively incorporate DHL into digital health interventions, there is a need to measure it with valid instruments and take into account the sociocultural and infrastructural impediments that define its manifestation in daily health behavior [11].

**Figure 2:**
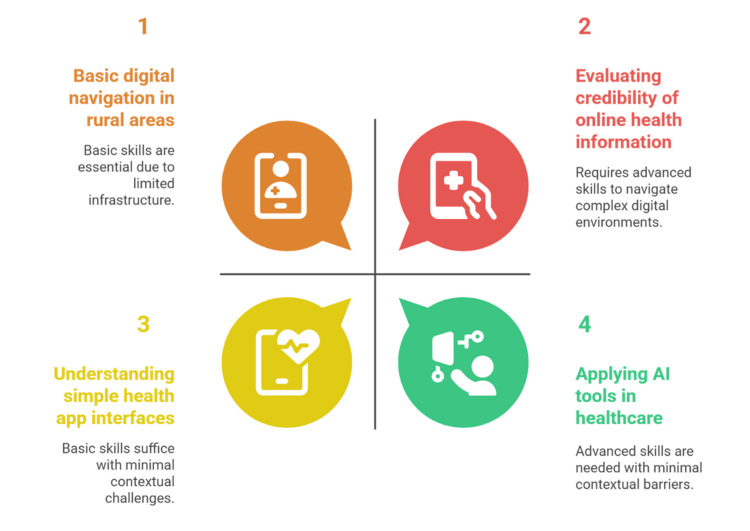
Contextual and skill-based variations in DHL requirements DHL, digital health literacy Figure created by the authors in collaboration with Napkin AI

Figure 2 depicts the different degrees of DHL needed to traverse healthcare technologies within the various sociocontextual environments.

Measurement tools for DHL

DHL quantitative measurement needs tools that can measure multidimensional skills in different user settings. The most commonly used of these is the eHEALS because it is short and easily administered [24]. Nonetheless, its use of self-perception and lesser coverage of interactive or social media-based platforms have created doubts about its scope and validity [25]. These gaps were filled by the creation of the DHLI, which included subjective and objective parts to assess functional skills like navigation, information appraisal, and data privacy [26]. DHLI is psychometrically sound and has been translated into different populations and settings [27].

Other instruments, for example, eHEALS-Extended, the Digital Literacy Scale, and the instruments that target LMICs, are expected to respond to the local needs by being more culturally specific and taking different forms [8]. Although performance-based approaches provide more accurate insights, they still lack scalability in low-resource settings because of the infrastructure limitations [28]. The lack of alignment between assessment methods and the abilities of the users, including linguistic mismatch or lack of experience with digital interfaces, can bias results in low-income populations. Self-report bias is especially strong in situations where the comprehension of a survey is impaired [29].

Combined measures of task-based measurement and self-report have demonstrated the potential to provide more realistic estimates. Notably, DHL measurement instruments are designed in the West and can incorporate assumptions regarding internet availability, digital trust, and content familiarity, which can be invalid in other contexts [21]. As such, contextually tailored instruments are necessary to prevent misclassification and inform equity-based digital health interventions. In the absence of such calibration, the need may be underestimated or the efficacy of the intervention overstated, which ultimately undermines the design and evaluation of the public health strategies in underserved communities [30].

Table 1 indicates that performance-based and culturally adapted instruments can provide a more precise and contextually specific measure of DHL than self-report instruments.

**Table 1:** Comparison of key DHL assessment instruments DHL, digital health literacy; DLS, Digital Literacy Scale; eHEALS, eHealth Literacy Scale; eHEALS-E, eHealth Literacy Scale-Extended; LMICs, low- and middle-income countries

Instrument	Format/items	Strengths	Limitations	Suitability for low-resource settings	Reference
eHEALS-E	Expanded version of eHEALS with added items	Incorporates social media and interactive use elements	Still relies on self-report; less validated in LMICs	Moderate	[2]
DLS	Objective performance-based tasks	Greater accuracy; reduces self-report bias	Lengthy; limited scalability	Low	[9]
Context-specific tools	Tailored to the cultural/digital context in LMICs	High contextual relevance can reflect linguistic and cultural nuances	Limited validation, often nonstandardized	High (but with trade-offs)	[13]
DHLI	Multidomain subjective + performance items	Captures broader DHL domains (e.g., privacy and content creation); adaptable	Resource-intensive; requires digital familiarity	High (with adaptation)	[16]
eHEALS	8-item Likert self-report scale	Widely validated; easy to administer	Measures perceived rather than actual skill; lacks coverage of social media or interactivity	Moderate	[24]

Socioeconomic determinants of digital access and literacy

Socioeconomic conditions are the main determinants of DHL and mediate access to digital tools as well as the capacity to utilize them effectively [5]. The most significant of these are income, education, geographic location, infrastructure, and sociocultural norms, which all converge to form unique DHL landscapes within different populations [16]. Income remains a major determinant of digital access. Higher-income earners have greater access to internet-connected devices, broadband subscriptions, and health-related applications, whereas low-income users tend to use shared devices or older models and prepaid data subscriptions, which restricts the consistency and privacy of their digital health experiences [31]. Such limitations drastically limit the possibilities of developing and implementing DHL in the preventive contexts. Education strongly predicts both general literacy and digital competencies. Higher education is linked with increased ease of using online systems, interpreting complicated health data, and the validity of online sources [32]. In contrast, individuals with low levels of formal education can have difficulties with medical terms, search engines, or abstract risk probability skills that are essential to participate in behaviors like screening or vaccination. Geographic disparities exacerbate these inequalities. Digital infrastructure in rural and urban slums is usually poor and unreliable, with poor internet connections, low electricity, and smartphone penetration [19]. Although the mobile-first approaches have increased the digital presence in most LMICs, technical access does not necessarily imply literacy or purposeful use. The affordability, speed, and stability of digital services continue to form fundamental issues in facilitating fair DHL development [2]. Gender roles and cultural norms also affect the acquisition and use of DHL. Women may have less agency over device use in some situations, gender-related ideas about health decision-making, or low self-efficacy in using digital tools independently [33]. Cultural barriers can also arise because of generational differences in digital exposure or trust in information on the Internet among older adults. These limit access and use of DHL in preventive health behavior. Language adds another layer of complexity. Most digital health tools are created in languages that are strong and national or international, leaving out speakers of indigenous or minor dialects [21]. Language barriers limit understanding and make it more dependent on intermediaries that compromise independence and accuracy of health decision-making.

More importantly, these socioeconomic and structural forces are not just moving DHL; they are defining the way the health systems ought to react. Interventions that overlook these determinants risk reinforcing existing inequities. As an example, a prevention program based on the assumption of universal access to smartphones or mastering the English language will not work in a low-income, multilingual rural area at all [34]. Structural solutions that can be used to mitigate these disparities should also be included in the interventions through the provision of device subsidies, offline-compatible applications, community-based training, and localized health content [3]. There is some potential that digital tools in combination with human support (e.g., health workers helping to use the app) can assist in overcoming both access and literacy barriers [24]. These methods represent a transition from user-blaming models of literacy to equity-based, systems-level DHL models, which acknowledge that skills cannot thrive in the absence of enabling environments [15]. The structural determinants of DHL are important to understand to develop inclusive preventive health interventions. As discussed in the following sections, DHL has a direct influence on the adoption of behaviors like screening, immunization, and personal hygiene. Nonetheless, such behavioral outcomes can be attained only in case digital environments are as inclusive and accessible as the messages that they convey.

Preventive health measures: scope and significance

Prevention is the key to population-wide management of disease and sustainable cost control in the health of the population. These are vaccination, early detection of noncommunicable diseases, hygiene behaviors including handwashing and sanitation, and lifestyle changes in diet, tobacco, alcohol, and physical inactivity [35]. The purpose of such interventions is not only to minimize the incidence of the disease but also to postpone its progression and complications and thereby enhance the quality of life and economic productivity. Empirical evidence supports their transformative impact. As an example, the Expanded Programme on Immunization has resulted in a 99% worldwide decrease in the occurrence of poliomyelitis [32], and well-organized breast and cervical cancer screening initiatives in high-income nations have been associated with a 20-30% decrease in deaths [13]. Community-based total sanitation campaigns are hygiene-based interventions that have reduced diarrheal disease burden by up to 36% in poorer areas [36]. Nonetheless, preventive uptake is not evenly distributed within and across countries, particularly in the socioeconomically disadvantaged groups. Only 22% of women of screening age in rural India receive cervical cancer screening, even though national campaigns are present [15]. Low health literacy, other livelihood needs, lack of trust in the health systems, and gender factors are some of the factors that still impede preventive participation in such environments [1]. Also, in most urban slums, preventive health is considered a luxury compared to other pressing health needs, and the logistical access to screening facilities is poor [36]. Prevention demands the cognitive and behavioral skills to interpret probabilistic risks, navigate health systems, and take action, which are beyond the realm of available information and agency of vulnerable groups [37]. DHL has become a major intervening factor in the ability of such people to access, analyze, and respond to preventive health information, particularly in technology-based interventions, as discussed in the following section.

Link between DHL and preventive behavior

Quantitative evidence is increasing that DHL is an important determinant of the uptake of preventive health behaviors [19]. The United States, Germany, and China studies indicate positive relationships between DHL and uptake of influenza vaccination, cancer screening, and hygiene practices [7]. As an example, the US cross-sectional survey reported that adults scoring high on DHL on the DHLI were 1.8 times more likely to have obtained a seasonal flu vaccine within the previous year [38]. Several mediating variables explain this relationship. To begin with, self-efficacy, or the personal confidence in the ability to perform a behavior, is enhanced as people can find and understand positive health information via the internet [3]. Second, digital technology trust has an immediate impact on the possibility of individuals using preventive apps or portals. In the Netherlands, a study showed that government-run health website trust was a better predictor of colorectal cancer screening participation than demographic factors [39]. Lastly, the health beliefs of perceived susceptibility and benefits are influenced by exposure to customized digital narratives and decision aids, which require DHL to correctly interpret themselves [14]. The DHL prevention connection in low-income settings is extraordinarily dependent on the presence of digital access as well as culture. A rural Kenyan study discovered that women who had access to mobile phones and had higher DHL were twice as likely to get prenatal checkups and tetanus vaccinations as their low-DHL counterparts [5]. Even in Brazil, where access to services was relatively equal, a positive relationship was found between DHL among the favela residents and COVID-19 vaccination adherence [40]. Conversely, limited DHL can neutralize otherwise effective programs. In the same state of Maharashtra, India, a cervical cancer screening SMS intervention did not increase attendance due to 40% of recipients being unable to read the messages or not seeing the relevance [26]. In a comparable manner, a voice-response-based tuberculosis screening program conducted in Dhaka faced low rates of participation because the users were unfamiliar with menu navigation and feared that their data would be misused [17]. These findings indicate that DHL not only determines access to digital information but also conditions its interpretation and transformation into preventive action. Bad DHL leads to reduced message salience, enhanced vulnerability to misinformation, and reduced behavioral follow-through. Thus, the effects of the digital public health campaigns cannot be evaluated completely without the data of the target populations.

Digital health interventions targeting vulnerable communities

There is a great variety of digital health interventions developed to increase access to preventive care by socioeconomically disadvantaged groups [8]. These are mHealth apps, SMS reminders, voice-response systems, and web-based self-assessment portals [41]. They are particularly attractive in settings where physical infrastructure is low, since they are relatively cheap, scalable, and able to convey information to individuals directly [10]. A number of documented programs have shown the potential and the constraints of such programs. India has had more than 130 million teleconsultations through the “eSanjeevani” platform, with a large number of those consultations involving follow-ups on preventive care in rural and tribal regions [42]. A large-scale South African program, “MomConnect,” targeting more than two million women, has enhanced the number of women receiving antenatal care and immunization by a factor of 25-35 with timed and simple SMS reminders [14]. Another randomized assessment of MomConnect showed that timely antenatal visits were higher in the intervention group by 28% compared to controls [11].

However, technology alone is insufficient. The lack of alignment with the DHL and contextual needs of the users is a major cause of failure of many digital health initiatives. Among 34 mHealth projects across sub-Saharan Africa reviewed, more than half were terminated within 18 months because of low usage or poor digital access by users [43]. As an example, in Bangladesh, the family planning app, called Taar Namti, experienced less than 10% continued use due to messages that were too complicated and not tailored to dialect or reading level [13]. The key factors in the success of interventions are language, interface design, and cultural relevance. Icon-based navigation, audio, and local language interventions are more effective than text-intensive and web-based interventions in low-literacy environments [44]. A malaria prevention app modified to include visual instructions and straightforward audio guidance was three times more engaged with in Ghana than its text-only predecessor [45].

Sustainability also remains a major concern. Projects that are donor-driven tend to have no continuity strategies or incorporation into national systems [6]. A meta-analysis of the mHealth programs in South and Southeast Asia found that only 14% of the initiatives were still running three years following the initiation of these projects because of low institutional involvement and a lack of evaluation standards [16]. The best programs are those that integrate DHL training into the intervention. In Rwanda, a maternal health application combined with group-based digital literacy training produced both better DHL scores and better compliance with taking iron supplements during pregnancy [17]. CHWs commonly serve as the crucial linking layer, translating the digital messages into culturally and linguistically specific versions for the final users [2]. Participatory design is also gaining traction. Co-creation with community members will allow for matching the content of the intervention, the delivery mode, and timing with the real-life restrictions and habits of the user [19]. In Nepal, a participatory video series (mobile video) on hygiene practices that was codesigned by female health volunteers resulted in a 33% increase in the use of household latrines [22]. Collectively, these illustrations show that digital interventions aimed at prevention in vulnerable communities are most effective when DHL is not considered a background factor but the main determinant of success. Interventions that assume DHL rather than building it may result in the amplification of the disparities. On the other hand, initiatives that put money into literacy, trust, and cultural alignment tend to produce lasting effects.

**Figure 3:**
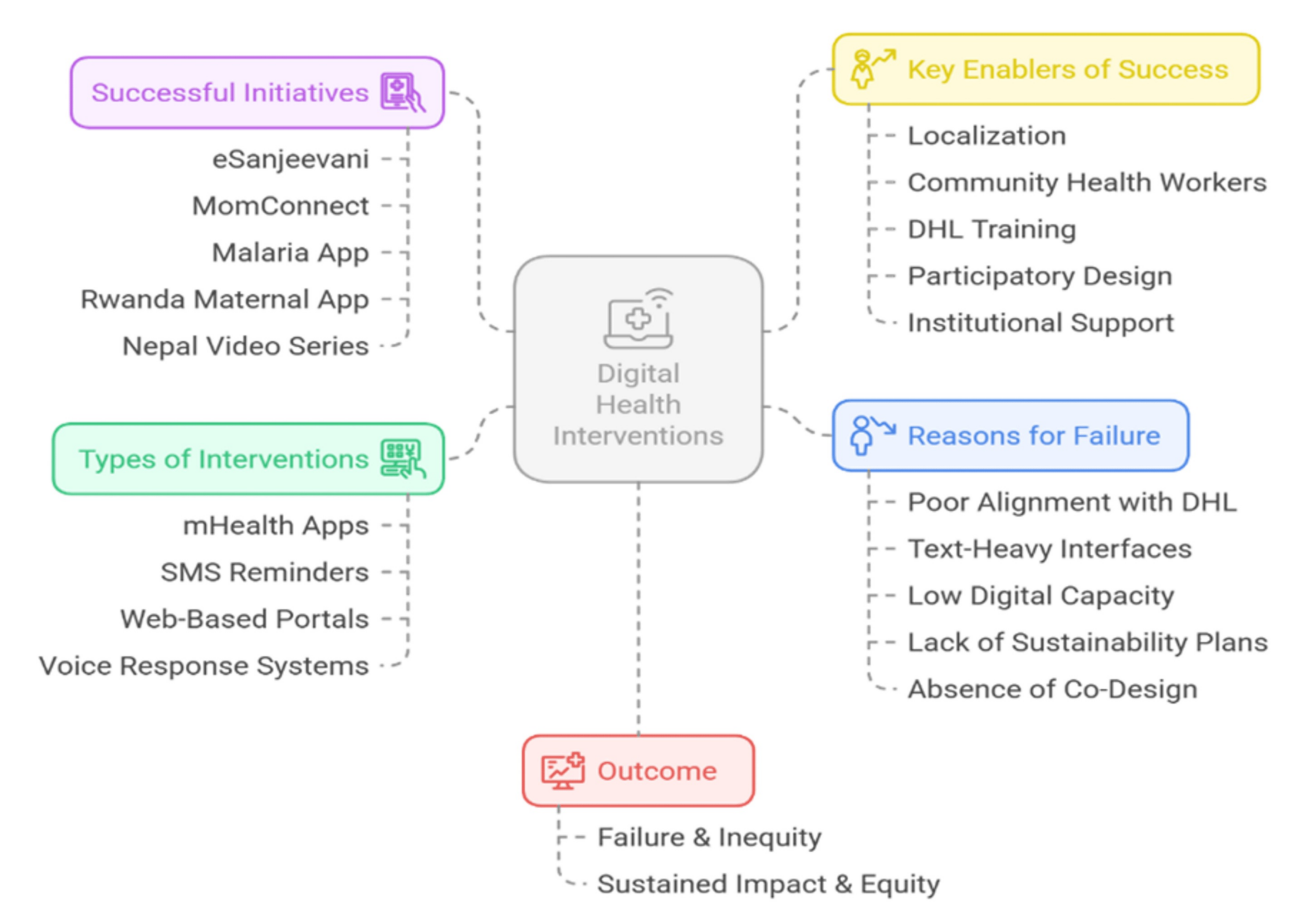
Determinants of success and failure in digital health interventions for vulnerable communities Figure created by the authors in collaboration with Napkin AI

Figure 3 shows the connection between the types of interventions, enabling factors, common barriers, and health equity outcomes.

Barriers to effective adoption of digital health tools

Although digital health tools can be used to change preventive health behavior, their effectiveness is usually constrained by a number of persistent barriers within the socioeconomically vulnerable populations [46]. Among the main obstacles are inadequate general literacy and inadequate health literacy, which grossly inhibit the process of understanding digital content, particularly when health information is presented in text-saturated interfaces or non-native languages [41]. This literacy deficiency is also exacerbated in areas where digital materials are not translated into local dialects or where guidelines are written using extensive formal medical language that cannot be interpreted by laypeople [1]. The other major obstacle is technology anxiety and mistrust of digital systems. Low-income and rural populations have many people who report feeling uncomfortable using mHealth applications due to concerns of making an error, surveillance, or revealing personal information online [32]. The credibility of digital content also depends on previous bad experiences with unreliable sources or fake news, which will prevent users from using a trustworthy platform. Such distrust is further compounded in societies where cultural beliefs and conventional health care oppose the mainstream digital recommendations [12]. The third type of impediment is caused by infrastructure restrictions, especially the uneven access to stable internet and electricity. Bandwidth restrictions, poor connectivity, and the absence of individual digital devices in remote and underserved areas make frequent interactions with health platforms unstable [47]. This issue of last-mile connectivity continues to reinforce digital exclusion, particularly among the older generation and women, who in many cases rely on shared or male-owned mobile devices [3]. The compounding impact of these obstacles compounds structural marginalization and limits access to preventive health information even where tools exist.

Role of health professionals and community workers

Health professionals and community health workers (CHWs) are an important point of connection between digital health systems and the populations they are intended to serve. They play a critical role in promoting DHL among low-resource populations whose trust in institutional health information could be weak [44]. Through community-based learning and demonstration of practical usage of digital platforms, CHWs can make technology less of a mystery to the users who are otherwise reluctant or even incapable of using these tools on their own [4]. Experience with various outreach programs, including the ASHA network in India and mTrac in Uganda, shows that CHWs are not only health educators but also agents of digital inclusion who help users learn to trust and take advantage of preventive health information [48]. These are the employees who often transform digital messages into oral, visual, or culturally acceptable messages, thus expanding the functionality of the mHealth apps off-screen [24]. Their position as both cultural brokers and information facilitators is particularly relevant in situations where health misinformation is pervasive and where social statuses or language exclusion inhibit the direct interaction of users and the platform [15]. Besides, CHWs can foster self-efficacy among marginalized users, such as guiding them through processes of downloading health apps, scheduling their appointments, or understanding risk calculators. The face-to-face scaffolding assists in encouraging long-term engagement and decreasing the dropout rates in digital health programs [49]. Notably, their participation also makes digital health interventions more credible since CHWs are usually integrated into their communities and enjoy a great deal of interpersonal trust. Therefore, any plan to improve DHL should incorporate training and incentivization of the health workers to serve as digital enablers within the preventive care ecosystems in a systematic way [20].

**Figure 4:**
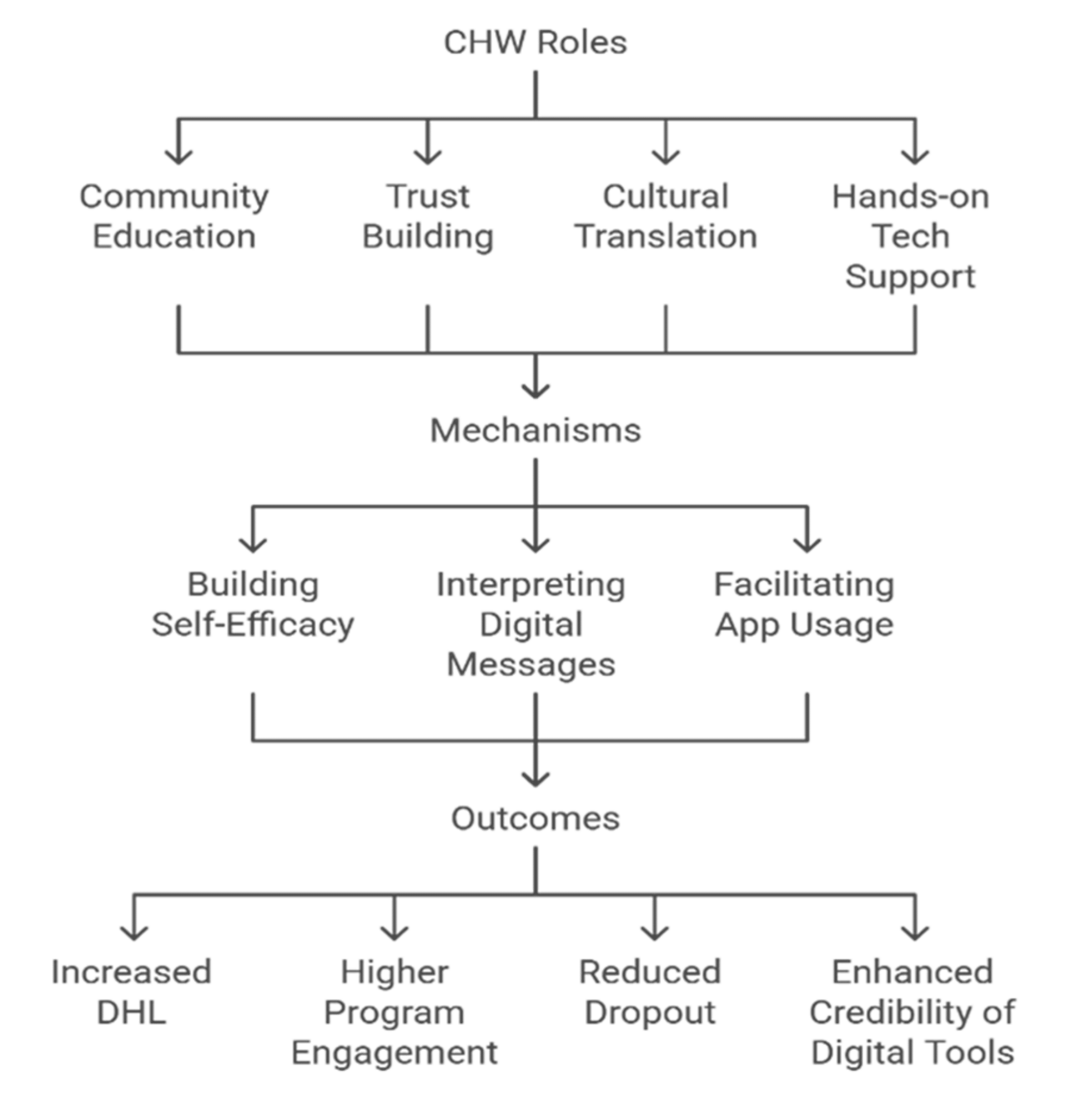
Role of CHWs in enhancing DHL and preventive care engagement policy and system-level enablers Figure created by the authors in collaboration with Napkin AI CHW, community health worker; DHL, digital health literacy

Figure 4 describes the process through which CHW-led education, trust-building, cultural translation, and tech support engage the mechanisms that enhance DHL outcomes and user engagement.

Although individual and community-level initiatives are the basis of improving DHL, they cannot be successful in the long term without national and global support systems [30]. An increasing number of countries, including India, Kenya, and Brazil, have started to integrate DHL into wider health and technological governance systems, recognizing that the behavior of individuals needs to be supported by institutional provision [27]. As an example, the National Digital Health Mission (NDHM) in India represents a dual interest in not only digitizing the housing of healthcare but also improving the capacity of the population to actively use their health data [6]. This agenda has been supported by WHO with the Global Strategy on Digital Health 2020-2025, which establishes DHL as a precondition of equity, access to health, and citizen-centered health engagement in the digital era [18].

Policy frameworks in isolation, however, cannot be used without the system-level mechanisms that can bring the DHL principles into daily health practices. Governments can make scalable approaches to delivering preventive care, which will be not only efficient but also inclusive through aligning digital innovations with the objectives of universal health coverage [7]. This will be more than just digital tools; it will need sustainable investments in interoperable technologies, cross-sectoral collaborations, and a regulatory ecosystem that is not only privacy-protective but also credible [49]. Equally important is the readiness of the health workforce. With structured digital training of frontline workers, they can be empowered to act not only as service providers but also as drivers of digital inclusion and behavior change [28].

The national health strategies integrating DHL at the systemic level make it impossible to increase existing health disparities through the digital transition [19]. Rather, when well governed, ethically designed, and inclusively implemented, DHL will serve as a driver of equitable preventive health among diverse populations.

Table 2 states that system-level enablers are key to integrating DHL into equitable preventive care.

**Table 2:** Strategic system-level enablers for advancing DHL and preventive health equity DHL, digital health literacy; NDHM, National Digital Health Mission

Strategic domain	Key enabler	Function	Reference
Workforce development	DHL training in public health curricula	Empowers frontline workers as community-level digital facilitators	[4]
Infrastructure expansion	Open-source health platforms and interoperable records	Reduces the digital divide; enables access for low-resource populations	[7]
Governance and regulation	Data privacy and misinformation oversight	Builds public trust; ensures ethical and secure digital environments	[14]
Collaborative investment	Public-private and philanthropic partnerships (e.g., Gates Foundation)	Supplements public infrastructure; scales DHL-focused interventions	[29]
Policy integration	WHO Global Strategy on Digital Health (2020-2025)	Links DHL to inclusion and universal health coverage via global frameworks	[38]
	NDHMs (e.g., India’s NDHM)	Embeds DHL in digital health policy and promotes citizen engagement in health data usage	[47]

Quantitative outcomes and gaps in current research

Empirical evidence is mounting to show a close relationship between DHL and the adoption of preventive health behaviors, particularly in resource-limited settings [9]. DHL does not only appear as a background variable but also as a key factor that determines whether people will or will not engage with preventive measures that include cancer screenings, human papillomavirus inoculations, dietary changes, and immunization compliance [50]. Higher DHLs tend to be more prepared to use such digital tools as SMS reminders, dietary tracking apps, or health portals; thus, they can be more likely to enjoy the benefits of preventive health innovations [11]. These trends suggest the presence of an equity gradient, in which populations with low DHL tend to be left behind in the digital health revolution, further widening disparities. Importantly, the success of the DHL-related interventions is largely increased when the material is locally adjusted and trust is transferred by means of CHWs or the public health authority [51].

However, the field is hampered by notable methodological inconsistencies. Most of the research is cross-sectional in design, making causal inference impossible, and longitudinal research that tracks long-term behavior change is uncommon [32]. The subjectivity of DHL measurement, with the use of subjective self-report instruments, such as the eHEALS [13], and performance-based indicators, makes it difficult to compare DHL measures across geographies and populations [52]. In addition, the current evidence is skewed toward HICs and has little to no representation of sub-Saharan Africa, Latin America, or marginalized groups in Asia and Oceania [53]. There is also little disaggregation by age, gender, or disability status, creating important knowledge gaps on how DHL affects preventive health behavior along intersecting identities [27].

Table 3 adds to this discussion by providing the geographic, methodological, and behavioral scope of the chosen empirical studies and pinpointing the most significant effect sizes in various settings.

**Table 3:** Empirical evidence linking DHL to preventive health behaviors across global contexts DHL, digital health literacy; eHEALS, eHealth Literacy Scale

Study location	Preventive behavior	DHL measure used	Study design	Key findings/effect size	Limitations	Reference
Global (various)	Multiple preventive actions (e.g., immunization and hygiene)	Mixed methods (eHEALS, DHLI, and task-based)	Meta-analysis	Medium to large effect sizes for DHL-based interventions	Potential publication bias; variation in outcome measures	[11]
Indonesia	Human papillomavirus vaccination (adolescents)	Task-based DHL assessment	Quasi-experimental	DHL improvement associated with a 20% increase in vaccine uptake	Sample restricted to urban adolescents; short follow-up period	[29]
Sub-Saharan Africa, Latin America, and Asia	Vaccination, diet, and screening	Varies (self-report and objective)	Mostly cross-sectional	The effectiveness of digital tools is significantly lower with low DHL	Regional underrepresentation; lack of longitudinal tracking	[32]
Brazil	Cancer screening	eHEALS	Cross-sectional	Individuals with high DHL were 3.6× more likely to undergo screening	Reliance on self-reported ability; limited causal inference	[40]

Limitations and future considerations

Although this review presents a strong synthesis of quantitative data on the correlation between DHL and preventive health behaviors in socioeconomically vulnerable groups, it has a number of limitations. To begin with, findings are limited in generalizability due to the heterogeneity of study populations, and most of them are confined to certain urban or rural areas in LMICs or HICs. The various socioeconomic, cultural, and infrastructural backgrounds restrict cross-comparison and extrapolation. Second, most studies were based on self-reported DHL and the adoption of behaviors, which created a risk of response bias, limitations of recall, and social desirability. The biases can either exaggerate or underrepresent the actual relationships between DHL and preventive practices. Besides, the variability of measurement tools in different studies, including nonuniform use of tools like eHEALS or DHLI, weakens effect size comparability and the interpretability of pooled evidence. Lastly, the majority of the available literature is cross-sectional, which precludes causal interpretations on the direction of the relationship between DHL and health behavior outcomes.

In the future, methodological and contextual gaps need to be filled in order to enhance the evidence base. Culturally and linguistically specific DHL interventions are urgently needed to support marginalized populations by being sensitive to the barriers that are specific to these communities, such as language diversity, technological anxiety, and local health beliefs. AI and adaptive digital platforms may allow more scalable and personalized health literacy solutions, especially when integrated into community-based health systems. Also, longitudinal cohort studies involving large and demographically diverse samples are needed to determine the long-term impact of DHL on preventive health outcomes. These designs will not only permit temporal inferences but also permit sustained behavior change over time to be monitored, providing a more rigorous basis for digital health policy and practice.

## Conclusions

This review highlights the importance of DHL as a key factor that determines preventive health behavior among the socioeconomically vulnerable. Although digital technologies like mHealth apps, SMS reminders, and online health portals have helped in increasing access to timely information, their effectiveness depends on the ability of the user to critically analyze and use digital information. According to quantitative studies, DHL is linked to an uptake of preventive services such as screenings and vaccinations by up to 25%, which is a significant contribution to the quality of the population. However, this potential remains unevenly distributed. Structural barriers to access, such as low levels of education, internet access, and fear of technology, which are misguided, further isolate the populations that are in the greatest need of preventive care.

Available literature has reported methodological loopholes, including unreliable use of validated DHL measures, overreliance on self-reported outcomes, and absence of longitudinal or interventional research that can allow causal inferences. What is more, DHL is usually presented as a fixed personal characteristic but not an evolving, socially constructed skill that depends on language and cultural relevance and digital confidence. DHL needs to be nurtured into the creation of equitable preventive care systems by codesigned, community-based interventions in line with the public health infrastructure. It is necessary to integrate DHL into more comprehensive equity-oriented programs, including national literacy programs and digital inclusion programs. Future research must focus on scalable, flexible DHL models that respond to the changing technologies and are based on lived community realities. It is only with such integrative approaches that digital health innovations can become tools of inclusion and not another means of exclusion.
